# PErineal Assessment and Repair Longitudinal Study (PEARLS): protocol for a matched pair cluster trial

**DOI:** 10.1186/1471-2393-10-10

**Published:** 2010-02-25

**Authors:** Debra E Bick, Christine Kettle, Sue Macdonald, Peter W Thomas, Robert K Hills, Khaled MK Ismail

**Affiliations:** 1 King's College London, Florence Nightingale School of Nursing and Midwifery, James Clerk Maxwell Building, 57 Waterloo Road, London, UK; 2 Faculty of Health, Staffordshire University, Blackheath Lane, Stafford, UK; 3 University Hospital of North Staffordshire, Maternity Centre, Stoke on Trent, Staffordshire, UK; 4 Royal College of Midwives, 15 Mansfield Street, London, UK; 5 Dorset Research and Development Support Unit, School of Health and Social Care, Bournemouth University, Royal London House, Christchurch Road, Bournemouth, Dorset, UK; 6 Department of Haematology, Cardiff University School of Medicine, Heath Park, Cardiff, UK; 7 Institute of Science and Technology, in Medicine, Keele University Medical School, Keele, Staffordshire, UK

## Abstract

**Background:**

The **Pe**rineal **A**ssessment and **R**epair **L**ongitudinal **S**tudy (PEARLS) is a national clinical quality improvement initiative designed to improve the assessment and management of perineal trauma. Perineal trauma affects around 85% of women who have a vaginal birth in the UK each year and millions more world-wide. Continuous suturing techniques compared with traditional interrupted methods are more effective in reducing pain and postnatal morbidity, however they are not widely used by clinicians despite recommendations of evidence based national clinical guidelines. Perineal suturing skills and postnatal management of trauma remain highly variable within and between maternity units in the UK as well as worldwide. Implementation of a standardised training package to support effective perineal management practices could reduce perineal pain and other related postnatal morbidity for a substantial number of women.

**Methods/Design:**

PEARLS is a matched pair cluster trial, which is being conducted in maternity units across the UK. Units within a matched pair will be randomised to implement the study intervention either early or late in the study period. The intervention will include the cascading of a multi-professional training package to enhance midwifery and obstetric skills in the assessment, repair and postnatal management of perineal trauma. Women who have had an episiotomy or second degree perineal tear will be eligible for recruitment. Prior to developing the intervention and deciding on study outcomes, a Delphi survey and a consensus conference were held to identify what women, who previously suffered perineal trauma during childbirth, considered to be important outcomes for them. Findings from this preliminary work (which will be reported elsewhere) and other outcomes including women's experiences of perineal pain and pain on activity, breastfeeding uptake and duration and psychological well-being as assessed using the Edinburgh Postnatal Depression Scale (EPDS) will be assessed at 10 days and three months post-birth.

**Discussion:**

Implementation of evidence-based perineal assessment and management practices, could lead to significantly improved physical and psychological health outcomes for women in the UK and world-wide.

**Trial registration:**

PEARLS is registered with the Current Controlled Trials Registry (no: ISRCTN28960026). NIHR UKCRN portfolio no: 4785.

## Background

Persistent perineal pain is one of the most commonly experienced health problems associated with birth [1,2]. It is a symptom highly related to perineal trauma and can impact on a woman's physical and psychological well-being as well as her relationship with her baby and family. Around 85% of women who have a vaginal delivery will sustain perineal trauma, which occurs either spontaneously or as a consequence of an episiotomy, and three-quarters of women will require suturing to facilitate healing of the disrupted tissue [3]. Studies investigating maternal morbidity have reported that for some women, perineal pain persists well beyond the postnatal period [1,2]. In the UK childbirth related perineal trauma is sub-divided into the following four types according to the tissues and structures involved (table 1). Third and fourth degree tears represent the most severe forms of perineal trauma, with a reported incidence of 0.5% to 3% [4], however, the actual incidence may be higher as some tears may be missed or misdiagnosed. Most childbirth related perineal trauma fall into the second-degree or episiotomy category.

**Table 1:** Classification of perineal trauma

**First degree**:	Injury to skin only
**Second degree:**	Injury to the perineum involving perineal muscles but not involving the anal sphincter
**Third degree:**	Injury to the perineum involving the anal sphincter complex; 3a < 50% of external anal sphincter (EAS) thickness torn; 3b > 50% of EAS thickness torn; 3c Internal anal sphincter (IAS) torn
**Fourth degree:**	Injury to perineum involving the anal sphincter complex (EAS and/or IAS) and anal epithelium

RCOG Green Top Guideline 23. Methods and Materials Used in Perineal Repair. Kettle C, O'Brien PMS.

### Suturing methods and materials

There are considerable variations in suture materials and techniques used for repairing perineal trauma in current clinical practice, despite guidelines and evidence to support best practice [5-9]. A Cochrane systematic review based on eight randomised controlled trials (RCTs) showed that the use of absorbable synthetic material is associated with less perineal pain and less requirement for analgesia [6]. A large RCT involving 1542 women compared the continuous and interrupted techniques for episiotomy and second degree perineal repair and reported a significant improvement in perineal pain with the continuous technique [7]. More recently, members of our collaborative team (CK, KMKI and RKH) conducted a Cochrane review of seven RCTs and reported that the use of continuous suturing techniques for all layers was associated with less short-term pain compared to the traditional interrupted method [8]. The RCOG Greentop and NICE intrapartum guidelines recommend that the continuous suturing technique and Vicryl Rapide suture material should be used for repair of episiotomy and second degree tears [5,9].

### Training issues

Identification, correct classification and the technique used to repair perineal injury are procedures that require knowledge and skills to ensure that the tissues involved are correctly aligned to facilitate healing and minimise postpartum morbidity. In the UK, the assessment and repair of trauma following vaginal delivery are mainly undertaken by midwives, some of whom may not have received adequate basic or updated training [10]. An audit of knowledge of perineal anatomy, classification of trauma and satisfaction with training in perineal management, which was undertaken by Sultan et al (75 midwives and 75 trainee doctors) reported that a large proportion of the participating clinicians had a poor understanding of perineal anatomy [10]. Only 20% of the trainees and 48% of midwives reported their training to have been of a 'good standard'. Many responses relating to questions on anatomy and perineal repair were incorrectly answered, for example, most classified a partial or complete tear of the EAS as 'second degree'. The anatomy of the perineum and pelvic floor and training in suturing is included within the pre-registration midwifery education programme, supported by practical experience in the clinical area. However, the time and focus allocated to this area of learning is variable between individual units and some midwives will not undertake perineal suturing until after qualifying when practical training usually takes place in the workplace. Moreover, there is little information to inform the content and delivery of training in perineal management and repair, which may only be undertaken at the instigation of the individual clinician.

### Postnatal care

In addition to gaps in immediate care, subsequent postpartum management is unlikely to be based on robust evidence or the degree of trauma sustained. Despite pain being experienced by hundreds of thousands of women in the UK, and many more worldwide and the availability of evidence-based guidance for postnatal management [3,11-13], the identification and management of longer-term perineal morbidity such as pain, dyspareunia, wound infection and haematoma, has not been a high priority in practice or research.

### Rationale for PEARLS

For the above reasons and in the absence of a standardised approach or formally recognised training programme for perineal management, our collaborative team felt that there was an urgent need to focus on the training needs of individual clinician as the unskilled operator, even when using the best materials and techniques, could contribute significantly to the extent of maternal morbidity. The main aim of this trial is to evaluate if enhanced clinician training in perineal assessment and management can reduce immediate and longer-term maternal morbidity, including perineal pain, and improve women's experiences of maternity care relating to the management of perineal trauma.

## Methods/Design

### Study design

The study is a multi-centre, matched pair cluster randomised trial. Matching criteria include unit size as determined by the total number of births per annum, type of unit (ie obstetric led or birth centre) and the qualifications of the facilitators, information on which were obtained from study sites on recruitment to the study. To ensure generalisability of the study findings, we will aim to recruit units of varying size, organisational structure and population demographics. A minimum of 20 units (10 pairs) will be recruited.

Prior to commencing the trial a Delphi survey and consensus conference were undertaken at two sites not participating in the main trial. The aim of this preliminary work was to generate a list of outcomes considered to be important by women who had recently experienced perineal trauma (≤ 6 months). A national survey of current midwifery clinical training and management in relation to childbirth-related perineal trauma was also conducted. The aim of the survey was to assess the level of implementation of national guidelines and reasons, if any, for lack of implementation prior to introducing any quality improvement intervention. The information generated from the survey will be a reference point to demonstrate the potential impact of the trial on the level of knowledge and clinical practice within maternity units in the UK. Results of the Delphi, consensus conference and national midwifery survey will be published elsewhere.

### Setting

The trial will be undertaken within a wide range of maternity units. Units will be analysed in matched pairs. An open invitation will be sent by the study team to Heads of Midwifery in maternity units across the UK exploring their intention to participate. Detailed information about the trial will be sent to units that show an initial intention to participate. Matching criteria parameters will be collected from each of the participating units.

### Participants/Eligibility criteria

All women who sustain a second degree perineal tear or episiotomy in a participating unit during the study period will be eligible. Women will not be eligible for recruitment if:

• under 16 years of age

• non-English speaking

• have suffered pregnancy loss

All births during a pre-specified period will contribute to analyses.

### Unit randomisation and collection of baseline data

**Figure 1:**
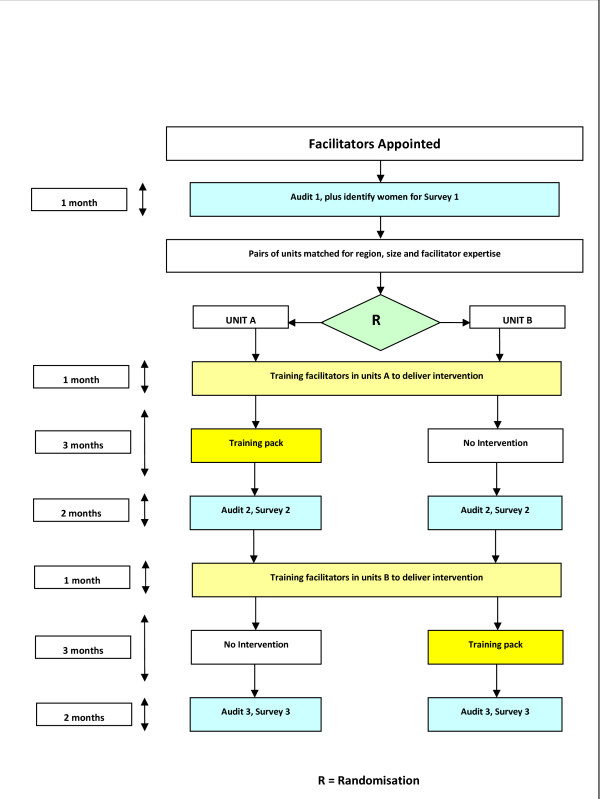
**Plan of investigation for matched pairs of units**.

Once pairs of units have been identified, third party randomisation will be carried out at Cardiff University; the statistician (RKH) will be blind to information that could identify the study unit. Following a baseline audit, one maternity unit from each matched pair will commence early implementation of the intervention. The use of a deferred training arm will allow primary study outcomes to be assessed between units in the pair at a second period of data collection and assessment of the sustainability of any improvements in those units randomised to early intervention. (see Figure 1).

All women booked for delivery in participating units will receive written information about the study during the antenatal period, at approximately 36 weeks gestation. Following the birth, all eligible women will be offered information about the study and given time to read the leaflet prior to being invited to take part. Informed consent will be obtained either on the Labour Ward, or prior to discharge home. Women who decide to participate will receive a study pack to take home, containing a covering letter, a 10-12 day questionnaire and a pre-paid reply envelope. A letter will also be sent to the GPs informing them of the study. Women who return the 10-12 day questionnaire will receive a second questionnaire and return envelope at three months postpartum.

### Intervention

The intervention that is being tested in the trial is a multi-professional training package to enhance the assessment and management of perineal trauma by midwives and obstetricians. The training package will comprise of:

▪ Reading material for independent study and self directed learning

▪ Copies of the RCOG and postnatal perineal care guidelines and perineal pain relief protocol [5,12,13]

▪ A formal workshop which will provide information on the principles of recognition and management of perineal trauma, surgical skills and simulated hands-on experience for the second degree & episiotomy repair

▪ An interactive CD-ROM to help participants refresh core information, and maintain competency

▪ An Objective Structured Assessment of Training (OSAT) proforma which will form part of the participants initial and ongoing assessment process, to be completed within 3 months of the training intervention

▪ A leaflet for all women who sustain sutured perineal trauma to promote self-management of their perineal trauma, and their general health and well-being, and advice on who to contact if they have any concerns

The intervention will take place during a three month period and will be delivered by local clinical facilitators. These will be clinicians from the unit (midwives or obstetricians) who have undergone intensive training in perineal assessment and management provided by the trial team, and will be responsible for cascading the training package within their units (with support from the project team). To ensure optimal recruitment of women from each study site, the project manager will maintain regular contact with the local clinical facilitators (at one to two weekly intervals) who will also have responsibility for overseeing recruitment at each site, following training in research procedures from the study team.

Delivering the intervention using local facilitators will accrue several benefits:

• It will ensure that the intervention could be delivered by a well trained clinician, not necessarily previously an expert in the field

• It will increase the sense of local ownership of the intervention within the participating units

• It will facilitate the use of Objective Structured Assessment of Training (OSAT)

• It will minimise potential confounding of an influential facilitator in that their level of experience will be one of the criteria for matching units

Facilitators will receive their training immediately prior to the implementation of the intervention in their unit. All other aspects of management will be as per local protocol.

### Data Collection

#### Baseline audit and surveys

Baseline data will be collected from each pair of units by undertaking a prospective clinical audit (Audit 1) over a period of around one month (although the precise length of time may be shorter, depending on the size of the units recruited).

The aim of the first audit (Audit 1) is to assess practice prior to any intervention, against quality standards for perineal management [5,12,13]. Women who give birth during the same time period, who have an episiotomy or sustain a second degree perineal tear, will be invited to complete questionnaires at 10-12 days (Survey 1A) and three months (Survey 1B) postpartum to monitor immediate and longer-term impact on health and other outcomes.

#### Audit and surveys following early intervention

Following implementation of the intervention in the units allocated to the early intervention group, a second prospective clinical audit (Audit 2) will be undertaken in both groups over a period of around two months. Women who give birth during the same time period, who have an episiotomy or sustain a second degree perineal tear, will be invited to complete questionnaires at 10-12 days (Survey 2A) and three months (Survey 2B) postpartum.

Data from Audit 2 and Surveys 2A-B will be compared to data from the previous audits and surveys (1A-B) within individual units to assess the impact that the intervention had in those units that receive the intervention, compared with those who had not so far received it. These data will inform the primary and secondary trial outcomes.

Following completion of this stage of the study, the local training facilitators in units randomised to later intervention will be trained to deliver the intervention in their individual units.

#### Later intervention period

Implementation of the intervention will commence in those units randomised to receive later intervention, delivered by local training facilitators with help and support from the central project team. The intervention will be delivered over a three month period.

#### Audit and surveys following late intervention

Following implementation of the intervention in units allocated to group B, a third prospective clinical audit (Audit 3) will be carried out in both groups over a period of two months. Women who give birth during the same time period, who have an episiotomy or sustain a second degree perineal tear, will be invited to complete questionnaires at 10-12 days (Survey 3A) and three months (Survey 3B) postpartum. Data from Audit 3 and Surveys 3A-B will be compared to data from the previous audits and surveys within individual units to assess the impact and sustainability of the implementation of the intervention.

### Outcome measures

#### Primary and secondary outcome

Previous studies of maternal morbidity have identifed several outcomes associated with perineal trauma, some of which will be used to measure improvements in maternal health following implementation of the intervention.

#### Primary Study Outcome

• Experience of perineal pain on daily activity at 10 - 12 days post birth.

#### Secondary Study Outcomes

10 - 12 days post birth

• severity of perineal pain

• need for suture removal

• use of pain relief duirng the previous 24 hours

• uptake and duration of exclusive breastfeeding

• perineal wound infection

Three months post birth

• Edinburgh Postnatal Depression Scale (EPDS [14]) score of ≥ 13

• timing of resumption of intercourse

• satisfaction with the perineal repair.

• duration of exclusive breastfeeding

### Data management and validation

Audit data for evaluation of the training package will be managed and validated locally, before being sent to the PEARLS Central Office. Survey participants will be identified and consented locally and the 10-12 day questionnaire and prepaid envelope will be given by ward staff prior to the woman's discharge from the postnatal ward. The three month questionnaire will be sent directly from the PEARLS Central Office, to women who consented and responded at 10-12 days. All completed questionnaires will be returned to the PEARLS Central Office, where data forms and electronic files will be securely stored (in locked filing cabinets or relational databases on password-protected computers). Data will be entered using form based entry systems, to ensure data quality. Automated data checking will be used to identify outliers and improbable data, and if data quality appears to be poor as a result of automated procedures, site data verification may be considered in a subset of centres.

#### Withdrawal

Only those women who sign a consent form and return the questionnaire at 10-12 days will be sent the three month questionnaire. Non-respondents will be deemed to have withdrawn. Where women respond at 10-12 days but not at three months, a reminder will be issued. If they fail to respond to the reminder they will be deemed to have withdrawn. In addition, participants may withdraw at any time by contacting the PEARLS Central Office. Reasons for withdrawal will be sought in this instance, but women will not be required to give details if they choose not to.

### Statistical issues

#### Sample size

In a clustered design, due consideration needs to be made of the fact that there are certain factors pertaining to the cluster (maternity unit), as well as to the individual, which can affect outcome measures, and hence the sample size depends not only on the size of difference one wishes to detect, but also the intra-cluster correlation coefficient (ICC) [15]. Typically such values are small, and it is very unlikely that the ICC will exceed 0.05. The sample size calculation for the trial assumes that at 10 - 12 days 75% of women have any pain whilst walking or sitting in the past 24 hours, that the ICC is 0.013, a 1% significance level, and a cluster size of 40. With 16 clusters (8 pairs) this would give the study 95% power to detect a 20% reduction in primary outcome from 75% to 55% (as seen in the trial by Kettle et al 2002 [7]) or alternatively a small-to-moderate difference of one third of a standard deviation on continuous scales, such as VAS scores for pain). This calculation assumes 0 correlation between paired clusters. Assuming a response rate of 60%, implies recruiting 67 women in each cluster. Having additional clusters will help to preserve sample size should clusters withdraw from the study.

### Data analysis

The analysis of this trial will be by 'intention to treat', in that we will attempt to include all clusters in the analysis regardless of implementation of the intervention, and attempt to get completed questionnaires returned from all women recruited in each cluster. A 5% 2-sided significance level will be used. Analysis of the primary and secondary outcomes, following the early intervention (i.e in phase 2), will be by means of the matched-pair random effects model [15] and analysed using a multi-level model framework [16] with MLWin software [17]. In this way the paired cluster design is taken into account. Results will be presented graphically as Forest plots to show the variation of effect size between cluster pairs. This framework permits extending the analyses to include individual level and paired-cluster level (such as cluster size, and baseline compliance with RCOG guidelines) variables as covariates. Multilevel models will be used to assess whether the effect of the intervention persists by looking in addition at the data following the second period of intervention. The proportion of eligible women who completed questionnaires will be calculated for each cluster and the intervention groups compared.

### Ethical considerations and Safety Committee

The protocol has been approved by the Thames Valley ethics committee. The conduct of the trial will be according to the principles of *MRC Guidelines for Good Clinical Practice in Clinical Trials *(1998) [18] and the appropriate NHS Research Governance Frameworks. All participating units will be required to sign an *Investigator's Agreement*, detailing their commitment to accrual, compliance, Good Clinical Practice, confidentiality and publication. Deviations from the agreement will be monitored and the Project Steering Committee will decide whether any action needs to be taken, e.g. withdrawal of funding, suspension of centre.

Site Specific R&D approval is required for each participating unit, whereby the NHS Trust Research and Development (R&D) Office will assess "locality issues" relating to the local population, investigators, facilities and resources. In order to comply with current arrangements, an employee of each unit (this could be the appointed local training facilitator) would have to assume responsibility as the local 'Principal Investigator', assisting the project team with procedures to obtain local R&D approval requirements for research governance. The PEARLS Central Office will help Principal Investigators obtain local R&D approval. The local Principal Investigator will be responsible for liaison with the local Trust management team regarding locality issues, and must obtain the necessary signatures from their Trust. Once local R&D approval have been granted and evidence provided to the PEARLS Central Office, the local Principal Investigator will receive a folder containing all the necessary materials to allow the trial to commence in their unit.

It is unlikely that the introduction of a training package in perineal care will cause any adverse reactions amongst women. However, any serious unexpected adverse events (SAEs) believed to be due to perineal suturing will be reported to the study office as soon as possible. This should be followed within 7 days by a completed SAE form. Events that might reasonably be expected to occur in women following childbirth do not need to be reported. For the purposes of this study, "serious" adverse events are those which are fatal, life-threatening, disabling or require hospitalisation, either for the mother, or the baby. "Unexpected" adverse experiences are defined as those that would not be expected among women following childbirth. An independent Data Monitoring and Ethics Committee (DMEC) will be convened, which will consider accumulating data from this and other studies, and advise the chair of the Trial Steering Committee if, in their view, there is both a) 'proof beyond reasonable doubt' that for all, or for some women or centres, the training package is definitely indicated or contra-indicated in terms of a net difference in the major endpoints, and b) evidence that might reasonably be expected to influence the management of women, the TSC can then decide whether to close or modify any part of the study. Unless this happens, however the central project team, the steering committee, the investigators and all of the central administrative staff (except the statisticians who supply the confidential analyses) will remain unaware of the interim results.

## Conclusions

This is the first RCT to quantify an evidence-based, 'hands on' training package to enhance the assessment and management of childbirth associated perineal trauma. It is likely to collect the largest data set to date on aspects of clinical management and women's health outcomes following birth related perineal trauma. A reduction in maternal morbidity as a consequence of enhanced clinical training has implications for practice and women's health globally.

## Competing interests

Financial competing interests

The Keele and Staffs Episiotomy Trainer was developed by Limbs and Things in collaboration with CK and KI through the Office of Research and Enterprise, Keele University and the University Hospital of North Staffordshire R&D Department. CK and KI receive a small royalty fee, paid via Keele University, from the sales of the trainer for their contribution. Other members of the PEARLS study group declare they have no financial or non-financial competing interests.

## Authors' contributions

DB and CK conceived the original study, and DB, CK, SM, RKH, KI and PT contributed to the study design. All authors edited the manuscript and read and approved the final manuscript.

## Pre-publication history

The pre-publication history for this paper can be accessed here:

http://www.biomedcentral.com/1471-2393/10/10/prepub
